# Recipient Age and Mortality Risk after Liver Transplantation: A Population-Based Cohort Study

**DOI:** 10.1371/journal.pone.0152324

**Published:** 2016-03-28

**Authors:** Hsiu-Pin Chen, Yung-Fong Tsai, Jr-Rung Lin, Fu-Chao Liu, Huang-Ping Yu

**Affiliations:** 1 Department of Anesthesiology, Chang Gung Memorial Hospital, Taoyuan, 333, Taiwan; 2 College of Medicine, Chang Gung University, Taoyuan, 333, Taiwan; 3 Clinical Informatics and Medical Statistics Research Center and Graduate Institute of Clinical Medicine, Chang Gung University, Taoyuan, 333, Taiwan; University of Toledo, UNITED STATES

## Abstract

The aim of the present large population-based cohort study is to explore the risk factors of age-related mortality in liver transplant recipients in Taiwan. Basic information and data on medical comorbidities for 2938 patients who received liver transplants between July 1, 1998, and December 31, 2012, were extracted from the National Health Insurance Research Database on the basis of ICD-9-codes. Mortality risks were analyzed after adjusting for preoperative comorbidities and compared among age cohorts. All patients were followed up until the study endpoint or death. This study finally included 2588 adults and 350 children [2068 (70.4%) male and 870 (29.6%) female patients]. The median age at transplantation was 52 (interquartile range, 43–58) years. Recipients were categorized into the following age cohorts: <20 (n = 350, 11.9%), 20–39 (n = 254, 8.6%), 40–59 (n = 1860, 63.3%), and ≥60 (n = 474, 16.1%) years. In the total population, 428 deaths occurred after liver transplantation, and the median follow-up period was 2.85 years (interquartile range, 1.2–5.5 years). Dialysis patients showed the highest risk of mortality irrespective of age. Further, the risk of death increased with an increase in the age at transplantation. Older liver transplant recipients (≥60 years), especially dialysis patients, have a higher mortality rate, possibly because they have more medical comorbidities. Our findings should make clinicians aware of the need for better risk stratification among elderly liver transplantation candidates.

## Introduction

Liver transplantation (LT) has become the routine treatment for patients with liver failure or end-stage liver disease. Because of advances in surgical techniques, anesthesia, infection control, critical care and immunosuppressants [1], the survival rate of LT recipients has greatly improved, and the number of LT patients has consequently increased, to include candidates previously considered too old or having too many medical comorbidities to receive LTs [2].

In particular, the number of older LT recipients has increased more rapidly than that of younger recipients, as an increasing number of healthy people are entering old age. Further, individuals aged <30 years may have a substantially lower risk of contracting hepatitis B virus (HBV) infection, which causes chronic hepatitis and consequently liver cirrhosis [3], may be substantially lower in aged <30 years because the Taiwanese government has enforced HBV vaccination since 1984 [4]. The association between age-related comorbidities and postoperative mortality risk remains a concern for LT recipients, especially the elderly ones [5,6], and careful evaluation is essential for elderly LT recipients, who potentially have more comorbidities. Preoperative assessment and long-term care has unique challenges in this particular transplant group [7]. Despite this, the recent contemporary literature lacks large population-based trials, and risk quantification and stratification among elderly LT recipients have not been analyzed yet.

In order to improve clinical practices, we analyzed all deaths after LT in Taiwan in the past 14.5 years with a focus on age-related risk factors of mortality. The present study aimed to assess the risk factors of pre-LT for mortality rate.

## Materials and Methods

### Data Collection

This was a retrospective national population-based cohort study by Taiwan’s National Health Insurance (NHI). The NHI program in Taiwan has gradually enrolled nearly 99.9% of the Taiwanese population (23.28 million). The Bureau of National Health Insurance (BNHI) has collected claim data in a de-identified and computerized format and established the National Health Insurance Research Database (NHIRD). This database provides registration files and original claim data from out- and inpatient care for reimbursement, including dates and orders of clinical visits and diagnostic codes from International Classification of Disease, Revision 9, Clinical Modification (ICD-9-CM). According to the NHI program, patients who need LT need to be diagnosed by transplant surgeons or gastroenterologists.

This study was evaluated and approved by the NHIRD research committee (NHIRD-103-103) and the institutional review board of Chang Gung Medical Foundation (103-0102B).

### Patient Definition and Selection

We identified LT patients from the catastrophic illness database between July 1998 and December 2012 using the ICD-9-CM codes V427 (LT status) and 996.82 (complications of transplanted liver). During this period, 4086 post-LT patients were registered in the NHIRD. LT recipients who did not undergo transplantation within this time period were not included in the present study; thus, 1148 patients who lacked a code for LT surgery (505, 75020A, or 75020B) were excluded, resulting in a final cohort of 2938 recipients.

Medical comorbidities were defined as five recorded outpatient department (OPD) diagnoses or one recorded inpatient department (IPD) diagnosis during the preoperative period. All diagnoses were verified using the configured ICD-9-CM codes. Further, the following comorbidities were defined using these codes: diabetes mellitus (ICD-9-CM 250), hypertension (ICD-9-CM 401–405), acute myocardial infarction (ICD-9-CM 410), congestive heart failure (ICD-9-CM 428), stroke (ICD-9-CM 430–438), peripheral vascular disease (ICD-9-CM 443), chronic pulmonary disease (ICD-9-CM 490–496), peptic ulcer (ICD-9-CM 533), chronic hepatitis (ICD-9-CM 571.4), liver cirrhosis (ICD-9-CM 571.5), psychosis (ICD-9-CM 295–299), cancer (ICD-9-CM 140–239), hepatic cellular carcinoma (ICD-9-CM 155), renal failure (ICD-9-CM 584–586) and infection (ICD-9-CM 038). Death was defined as the termination of national health insurance or receipt of insurance death codes.

### Measurements

The primary outcome was long-term mortality in the LT recipients. The survival time of the LT recipients was calculated as the date of surgery to the date of death. Variables used to estimate the risk of mortality included demographic and clinical characteristics, such as age, gender, and comorbidities.

### Statistical Analysis

The chi-square test or Fisher’s exact test was used to examine differences in demographic characteristics among different age cohorts. Risk ratios were used to compare the transplant cohort with the general population. Kaplan—Meier estimates with log-rank tests were used to compare survival during follow-up among the age cohorts. In the mortality analyses, patients were followed up until an event (death) or censoring (loss to follow-up or end of the follow-up period), whichever occurred first. All statistical tests were two-sided, and a *p*-value < .05 was considered statistically significant. Cox proportional hazards regression modeling was used to analyze the effect of age-related mortality, when modeled as a continuous and as a categorical variable, to predict the age as a risk factor after risk adjustment. Analyses were performed using SAS statistical software (version 9.3; SAS Institute Inc.; Cary, NC, USA).

## Results

### Study Population and Baseline Characteristics

A total of 2938 LT procedures in 2588 adults and 350 children were performed and recorded in the Taiwanese NHIRD during the study period. The overall median age at the time of transplantation was 52 years (interquartile range: 43–58 years), and the study included 2068 (70.4%) male and 870 (29.6%) female patients. Recipients were categorized into the following age cohorts: <20 years (n = 350, 11.9%), 20–39 years (n = 254, 8.6%), 40–59 years (n = 1860, 63.3%), and ≥60 years (n = 474, 16.1%).

Older patients were more likely to have hypertension, diabetes mellitus, coronary arterial disease, chronic pulmonary disease, heart failure, stroke, and liver cirrhosis than young patients. Moreover, chronic hepatitis was the most common medical comorbidity and was recorded in 2702 (91.97%) patients. Table 1 shows the significant differences between the basic characteristics among the age groups.

**Table 1:** General demographics of liver allograft recipients.

Variable	Age groups	<20	20–40	40–60	≥60	Total	*p*-value
**Number (%)**		350	254	1860	474	2938	
**Gender**	Male	173 (49.43)	191 (75.20)	1432 (76.99)	272 (57.38)	2068 (70.39)	< .0001
	Female	177 (50.57)	63 (24.80)	428 (23.01)	202 (42.62)	870 (29.61)	
**Medical comorbidity**	HTN	1 (0.29)	12 (4.72)	398 (21.40)	187 (39.45)	598 (20.35)	< .0001
	Pulmonary disease	34 (9.71)	17 (6.69)	263 (14.14)	106 (22.36)	420 (14.30)	< .0001
	DM	1 (0.29)	15 (5.91)	458 (24.62)	140 (29.54)	614 (20.90)	< .0001
	Stroke	2 (0.57)	4 (1.57)	53 (2.85)	32 (6.75)	91 (3.10)	< .0001
	CAD	3 (0.86)	2 (0.79)	114 (6.13)	83 (17.51)	202 (6.88)	< .0001
	CHF	1 (0.29)	2 (0.79)	29 (1.56)	17 (3.59)	49 (1.67)	0.0018
	CKD / ESRD	1 (0.29)	7 (2.76)	76 (4.09)	25 (5.27)	109 (3.71)	< .0001
	Dialysis	1 (0.29)	2 (0.79)	24 (1.29)	4 (0.84)	31 (1.06)	0.3411
	Liver cirrhosis	155 (44.29)	175 (68.90)	1613 (86.72)	415 (87.55)	2358 (80.26)	< .0001
	Chronic hepatitis	221 (63.14)	218 (85.83)	1781 (95.75)	458 (96.62)	2678 (91.15)	< .0001
	Alcoholic hepatitis	0 (0.00)	63 (24.80)	425 (22.85)	58 (12.24)	546 (18.58)	< .0001
	HBV	4 (1.14)	108 (42.52)	962 (51.72)	189 (39.87)	1263 (42.99)	< .0001
	HCV	0 (0.00)	0 (0.00)	19 (1.02)	6 (1.27)	25 (0.85)	0.0597
	Peptic ulcer	8 (2.29)	84 (33.07)	1069 (57.47)	271 (57.17)	1432 (48.74)	< .0001
	Psychosis	0 (0.00)	20 (7.87)	267 (14.35)	98 (20.68)	385 (13.10)	< .0001
	Esophageal varices	70 (20.00)	89 (35.04)	712 (38.28)	148 (31.22)	1019 (34.68)	< .0001
**Cancer**	HCC	8 (2.29)	61 (24.02)	812 (43.66)	255 (53.80)	1136 (38.67)	< .0001
	Non-HCC	13 (3.71)	15 (5.91)	164 (8.82)	52 (10.97)	244 (8.30)	0.0008

Chi-square test or Fisher's exact test were used to examine the differences in the demographic characteristics of liver transplant patients between the age cohorts. HTN = hypertension, DM = diabetes mellitus, CAD = coronary artery disease, CHF = congestive heart failure, CKD / ESRD = chronic kidney disease/end stage renal disease, HBV = chronic B hepatitis infection, HCV = chronic C hepatitis infection, HCC = hepatocellular carcinoma.

### Data Accuracy

In order to verify the quality of the data in the NHIRD, we extracted the number of patients receiving LTs from the Taiwan Transplant Database. From 2005 to 2012, 2775 LTs were registered in the Taiwan Transplant Database, and during the same period, the number in the NHIRD was 2723, indicating a small discrepancy between these databases (concordance: 98.1%).

### Mortality Risk

**Fig 1:**
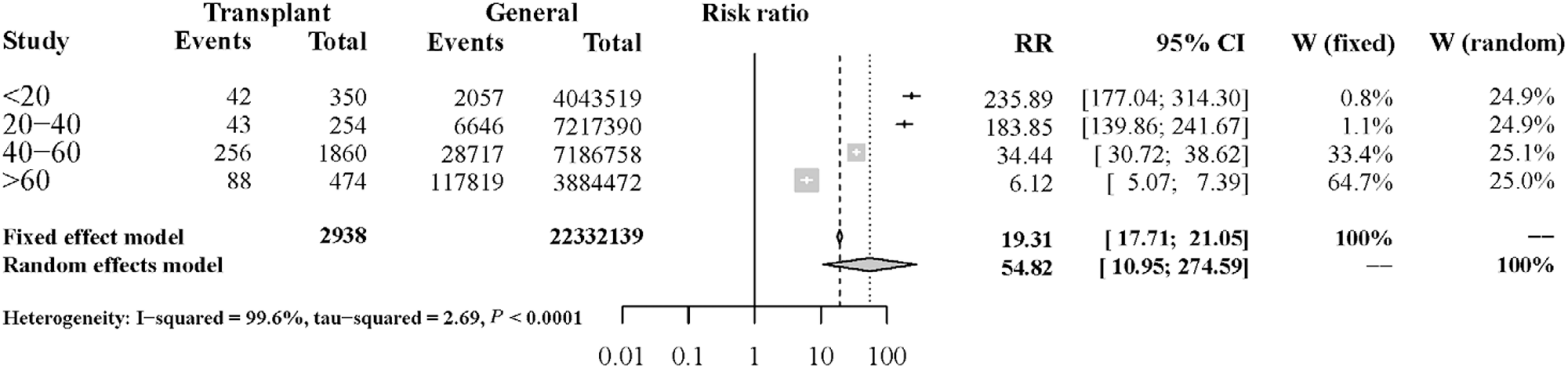
Mortality risk ratios of liver transplant recipients versus the general population.

Fig 1 shows a comparison of mortality rates between the transplant population and the general Taiwanese population during the same time period according to age group. The rates were significantly higher in the transplant population than in the general population.

**Fig 2:**
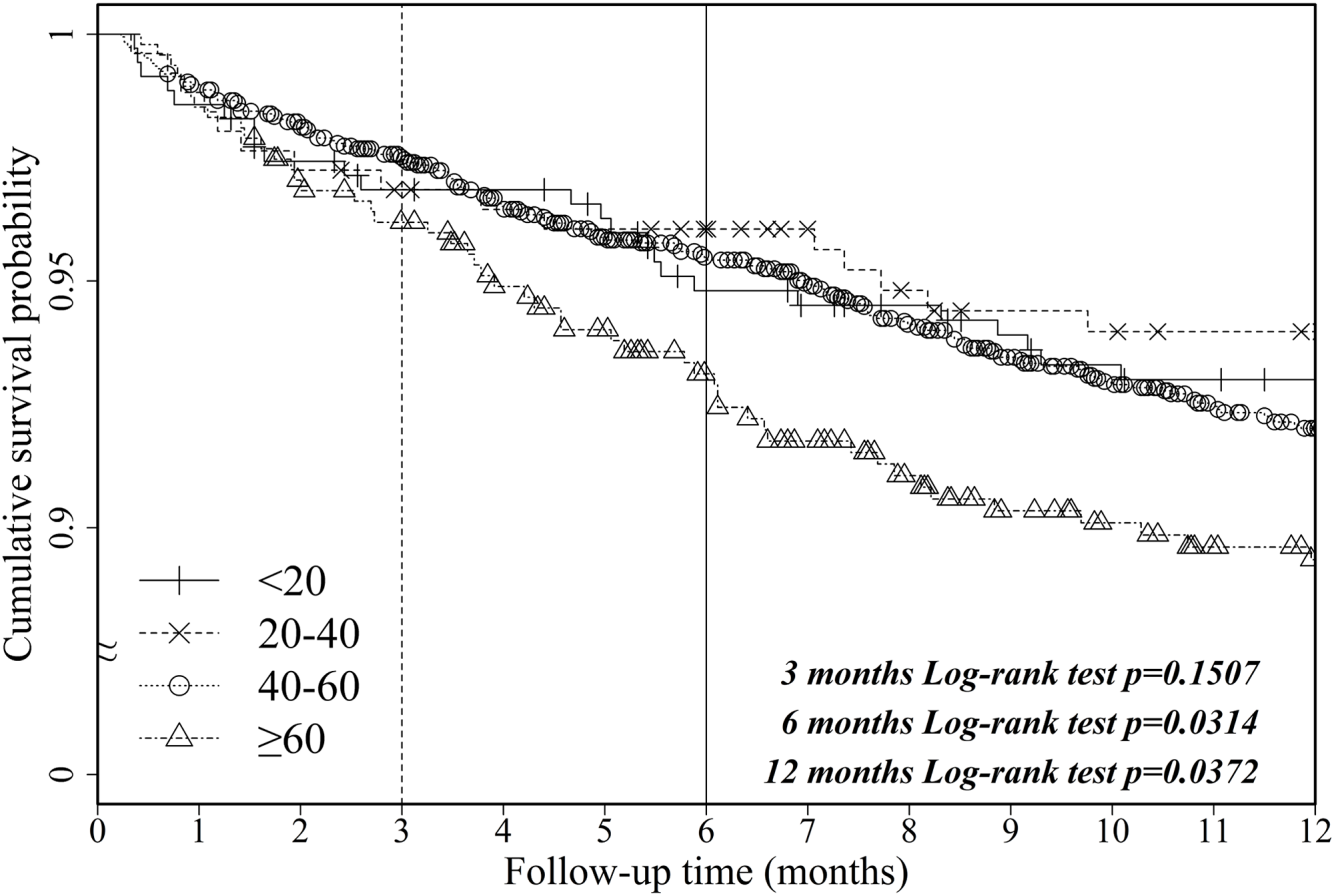
Unadjusted Kaplan—Meier survival curves of first-year patient survival after LT in the past 14.5 years among liver transplant recipients stratified by age cohort.

**Fig 3:**
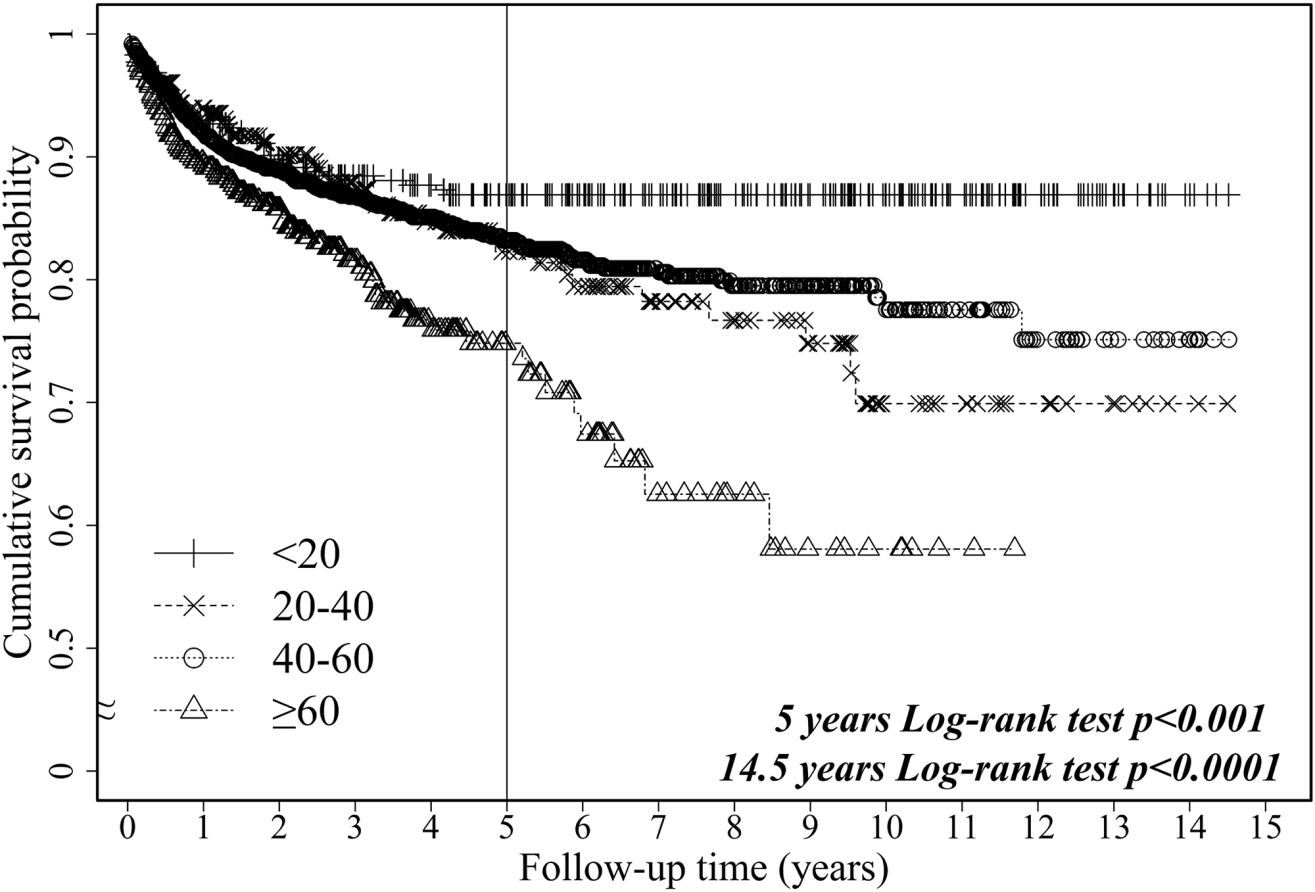
Unadjusted Kaplan—Meier survival curves of risk of mortality after liver transplantation in the past 14.5 years stratified by age cohort.

Figs 2 and 3 present the unadjusted Kaplan—Meier survival curves for first-year patient survival and the risk of death after LT during the 14.5-year study period, respectively, according to recipient age. The six-month and first-year patient survival rates were similar between the <20 years, 20–40 years, 40–60 years, and ≥60 years groups, showing only a slightly significant difference (Log-rank *p* = .0314 and *p* = .0372); however, the five-year and during the study period patient survival rates in these groups were significantly different (Log-rank *p* < .001 and *p* < .0001). These findings indicated that survival was strongly related to age at the time of transplant, with older patient groups showing higher mortality.

**Fig 4:**
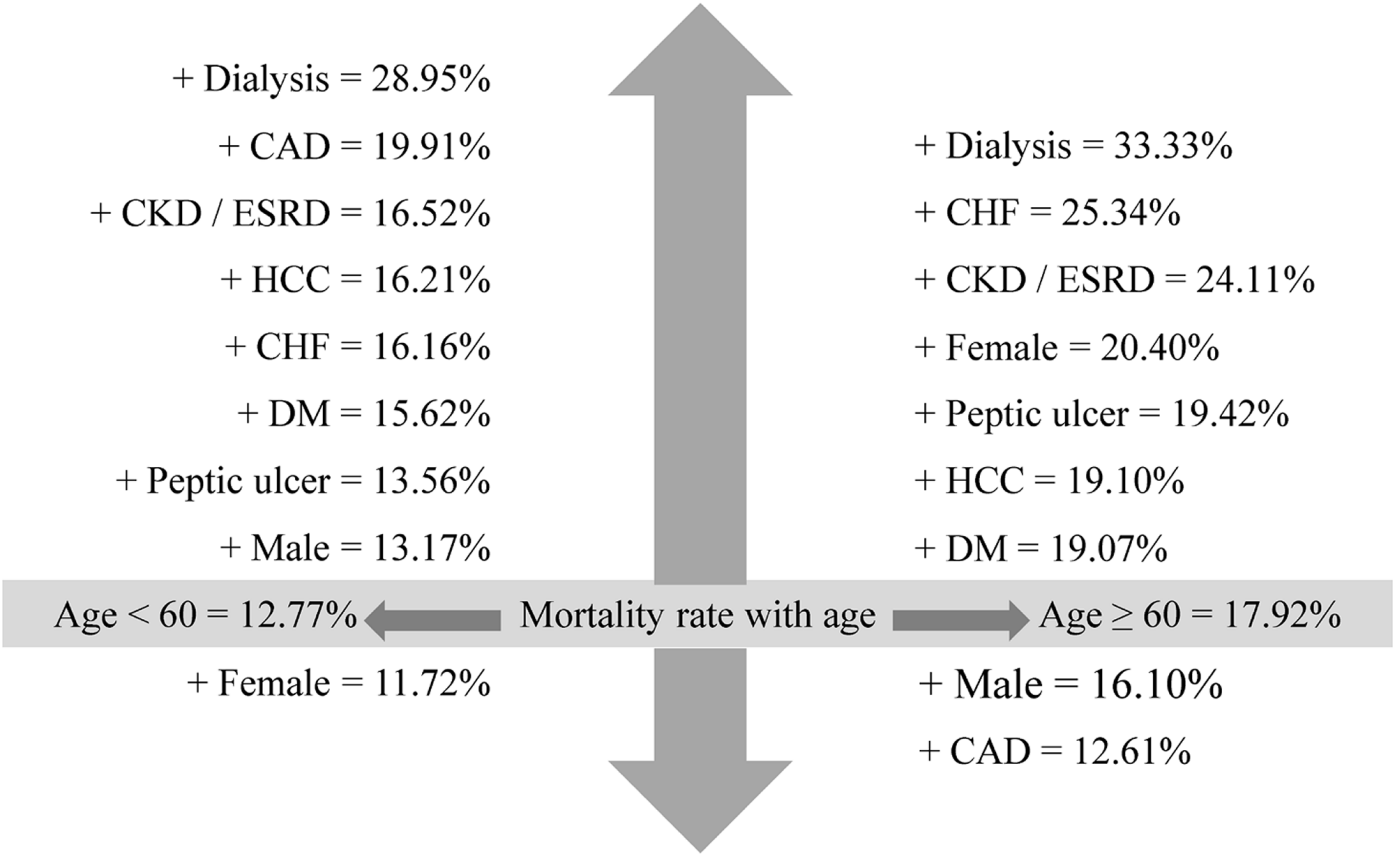
Risk of death among liver transplant recipients aged <60 or ≥60 years during a median follow-up period of 2.85 years posttransplantation with risk adjustments for the presence of a single additional covariable at the time of transplantation. DM = diabetes mellitus, CAD = coronary artery disease, CHF = congestive heart failure, CKD/ESRD = chronic kidney disease/end-stage renal disease, HCC = hepatocellular carcinoma.

This study identified 342 deaths that occurred posttransplantation during a median follow-up period of 2.85 years (interquartile range: 1.2–5.5 years). Fig 4 shows the changes in mortality at the cut-off age of 60 years, which was selected arbitrarily. The risk of death was 17.92% among liver transplant recipients aged ≥60 years during a median follow-up of 2.85 years posttransplantation, and this metric changed substantially depending on the presence of additional factors. For instance, dialysis, congestive heart failure, chronic kidney disease/end-stage renal disease, and peptic ulcer were significantly associated with the high mortality rate in transplant recipients aged ≥60 years. On the other hand, female gender was significantly associated with a low mortality rate in LT recipients aged <60 years. The most influential additional risk factor of mortality in liver transplant recipients irrespective of age was renal dialysis (the mortality rate increased from 12.77% to 28.95% in the <60 year cohort and from 17.92% to 33.33% in the ≥60 years cohort). Besides that we also analyzed the incidence of infection post LT. The incidences of infection were close between <60 years (n = 147, 6.0%) and ≥60 years (n = 18, 3.8%) groups, showing no significant difference (*p* = .060).

Cox proportional hazard model was used to adjust age groups. Table 2 shows the impact of the age groups (≥60 years vs. <60 years) remained significant after adjusting risk factors including dialysis, diabetes mellitus, coronary artery disease, congestive heart failure, chronic kidney disease, peptic ulcer and hepatocellular carcinoma (hazard ratio, 1.475, 95% confidence interval, 1.154 to 1.887; *p* = .0020). We also assessed the model by stratified the patients into three risk groups (the number of comorbidities: none, one, over one). At none comorbidity group, the impact of the age groups is significant (hazard ratio, 2.374, *p* = .0039), and at more than one type of comorbidity group, the impact of the age groups is also significant (hazard ratio, 1.425, *p* = .0310).

**Table 2:** Multivariable Cox proportional hazard model for age-related mortality after transplantation.

**Variable**	**Hazard Ratio**	**95% CI**	***p*-value**
Age ≥ 60 v.s. < 60*	1.475	1.154–1.887	0.0020
Dialysis	2.083	0.985–4.405	0.0547
DM	1.107	0.876–1.399	0.3929
CAD	1.177	0.816–1.698	0.4424
CHF	1.218	0.626–2.368	0.5617
CKD	1.210	0.744–1.969	0.4424
PU	1.118	0.918–1.361	0.3929
HCC	1.400	1.149–1.705	0.0009
**The model was stratified by the risk groups (the number of comorbidities)**			
**Age-related (comorbidities)**	**Hazard Ratio**	**95% CI**	***p*-value**
Age ≥ 60 v.s. < 60 (none)	2.374	1.320–4.270	0.0039
Age ≥ 60 v.s. < 60 (one)	1.327	0.852–2.066	0.2104
Age ≥ 60 v.s. < 60 (over one)	1.425	1.033–1.967	0.0310

CI = confidence interval; DM = diabetes mellitus, CAD = coronary artery disease, CHF = congestive heart failure, CKD = chronic kidney disease, PU = peptic ulcer, HCC = hepatocellular carcinoma.

*Model adjusted for risk factors including dialysis, DM, CAD, CHF, CKD, PU and HCC.

## Discussion

Considering associated medical comorbidities and clinical outcomes is essential while formulating policies on organ transplant allocation. There are several concerns related to older LT recipients, including whether they have similar survival rates as their younger counterparts. To our knowledge, the present study is the first to examine data from a large database and determine whether elderly LT recipients have significantly higher overall mortality rates.

From the NHIRD, we found that an increasing number of elderly patients are undergoing LT surgery [1,7]. The results of the present study also showed that overall survival is low in elderly LT recipients. The mortality rates of recipients aged ≥60 years in the transplant cohort were compared with those of individuals aged ≥60 years from the general Taiwanese population during the same time period, and significantly higher mortality rates were observed in the transplant population. Thus, age was found to be a major risk factor for post-LT mortality, indicating that elderly LT recipients must be properly screened according to the presence or absence concomitant risk factors (including age and comorbidities) [8–12]. We recommend that future studies focus on elderly LT patients, which may supply clinicians with data for better risk stratification among elderly LT candidates.

Renal insufficiency is an important indicator of post-LT mortality and morbidity. Irrespective of age, patients undergoing dialysis in the present study showed a higher LT mortality rate. Renal replacement therapy before LT has been found to have a strong correlation with poor short- and long-term patient survival among LT patients [13–15]. Further, renal dysfunction before LT surgery has been reported to make transplant recipients susceptible to bacterial or fungal infections [16,17], posttransplant sepsis, prolonged intensive care unit stay [18], requiring renal replacement therapy [19], and greater overall cost of treatment [20,21].

In our study, after adjusting major risk factors, the impact of the age remained significant. Besides that, older LT patient with more medical comorbidities, the impact of the age is also significant. Therefore, what we need to do is optimizing the selection of elderly patients, this might minimize the difference. With the same indications of younger LT patients, we should evaluate elderly patients with good functional status without significant medical comorbidities for transplantation [22]. If, the elderly become too ill, the best way is to advise from transplant. Because death followed prolonged post-LT hospital course is more shattering than death itself, especially if this could be predicted before LT.

The present large retrospective population-based cohort study does have some limitations. Although the National Health Insurance Bureau regularly cross-checks each hospital’s claims in order to reduce coding infractions and diagnoses and coding in hospitals and NHIRD services are generally considered accurate, the data are still susceptible to human errors, for example, inaccurate coding [23]. In addition, the NHIRD is a secondary database and lacks actual test data, including the physical examination and laboratory findings of patients, specific etiological data for end-stage liver disease leading to LT, and models for end-stage liver disease scores, which are related to patient mortality [24–26]. The NHIRD database is lack of the cause of death and allograft loss. Since cause of death and allograft loss were not analyzed, it remains to be the limitation of the study. Finally, although this population-based cohort study shows the relationship between age, comorbidities, and mortality among LT recipients, precise causality remains unknown [5,6,27,28].

## Conclusion

Liver transplant recipients aged ≥60 years show higher mortality rates compared to similarly aged individuals from the general population as well as LT recipients aged <60 years. This increased mortality seems to be associated with medical comorbidities, especially in dialysis patients. We believe that our findings should not be a factor preventing elderly candidates from receiving transplants; rather, they should enable clinicians to perform better risk stratification for elderly transplant candidates. We also recommend that further prospective studies be conducted to understand how age and comorbidities affect mortality in elderly LT recipients.

## Supporting Information

S1 Fig Number of recepients after liver transplantation. (XLSX)

S2 Fig First year mortality of transplant patients. (XLSX)
